# Supplementary material for: Genetic Testing to Predict Weight Loss and Diabetes Remission and Long-Term Sustainability after Bariatric Surgery: A Pilot Study
Source: J Clin Med. 2019 Jul 3;8(7):964. doi: 10.3390/jcm8070964 (PMC6679039; doi:10.3390/jcm8070964)
Supplement: Supplementary file 1 [file jcm-08-00964-s001.pdf]

Supplementary Materials

GPSs evaluated in the study and the risk score calculation.

GPS: genetic prediction risk score. SNP: single nucleotide polymorphism. GPSs are calculated by using an additive model based on the total number of risk alleles in each genotype of the assessed SNP.

GPS diabetes

SNP	GENE	Minor Allele	Major allele	Risk allele	Protective allele	Risk homozygote	Risk heterozygote
rs4343	ACE	G	A	G	n.a.	2	1
rs16861209	ADIPOQ	A	C	A	n.a.	2	1
rs5186	AGTR1	C	A	C	n.a.	2	1
CD010	APOC3	A	G	A	n.a.	2	1
rs7754840	CDKAL1	C	G	C	n.a.	2	1
rs10811661	CDKN2B	C	T	C	n.a.	2	1
rs696217	GHRL	T	G	T	n.a.	2	1
rs1800795	IL6	C	G	n.a.	C	-2	-1
rs12970134	MC4R	A	G	A	n.a.	2	1
rs1800206	PPARA	G	C	G	n.a.	2	1
rs1801282	PPARG	G	C	C	n.a.	2	1
rs7903146	TCF7L2	T	C	T	n.a.	2	1
rs10010131	WFS1	A	G	n.a.	A	-2	-1
CD014	PON1	T	A	A	n.a.	1	0

GPS Appetite regulation

SNP	GENE	Minor Allele	Major allele	Risk allele	Protective allele	Risk homozygote	Risk heterozygote
rs6265	BDNF	A	G	G	n.a.	2	1
rs925946	BDNFOS	T	G	T	n.a.	2	1
rs12535708	LEP	A	C	C	n.a.	2	1
rs52820871	MC4R	G	T	n.a.	G	-2	-1
rs17700633	MC4R	A	G	A	n.a.	2	1
rs2229616	MC4R	A	G	n.a.	A	-2	-1

rs10838738	MTCH2	G	A	G	n.a.	2	1
rs4580704	CLOCK	G	C	C	n.a.	1	0
rs4864548	CLOCK	A	G	A	n.a	2	1
rs17782313	MC4R	C	T	C	n.a	2	1

GPS- Weight loss in response to exercise

SNP	GENE	Minor Allele	Major allele	Risk allele	Protective allele	Risk homozygote	Risk heterozygote
rs328	LPL	G	C	n.a.	G	2	1
rs696217	GHRL	T	G	n.a.	T	2	1
rs4994	ADRB3	C	T	n.a.	C	2	1
rs1800795	IL6	C	G	n.a.	C	2	1
rs9693898	Chr.8	G	A	G	n.a.	2	1

GPS weight loss in response to diet

SNP	GENE	Minor Allele	Major allele	Risk allele	Protective allele	Risk homozygote	Risk heterozygote
rs2419621	ACSL5	T	C	n.a.	T	2	2
rs5082	APOA2	T	C	n.a.	C	2	0
rs651821	APOA5	C	T	n.a.	C	2	1
rs894160	PLIN1	A	G	n.a.	A	2	1
rs1137100	LEPR	G	A	n.a.	A	2	0
rs1800849	UCP3	T	C	n.a.	C	2	0
rs659366	UCP2	T	C	n.a.	T	2	1
rs1801282	PPARG	G	C	G	n.a.	-2	-2
rs6824447	Chr.4	G	A	G	n.a.	2	1
rs1052700	PLIN	A	T	n.a.	A	2	0

GPS-life style interventions (Moleres)

SNP	GENE	Minor Allele	Major allele	Risk allele	Protective allele	Risk homozygote	Risk heterozygote
rs9939609	FTO	A	T	A	n.a.	2	1
rs17782313	MC4R	C	T	C	n.a.	2	1
rs1800795	IL-6	C	G	C	n.a.	2	1
rs1801282	PPARG	G	C	G	n.a.	2	1
rs2241766	ADIPOQ	G	T	T	n.a.	2	1

GPS-bariatric surgery (Still)

SNP	GENE	Minor Allele	Major allele	Risk allele	Protective allele	Risk homozygote	Risk heterozygote
rs7566605	INSIG2	C	G	C	n.a.	2	1
rs9939609	FTO	A	T	A	n.a.	2	1
rs17782313	MC4R	C	T	C	n.a.	2	1
rs6235	PCSK1	C	G	C	n.a.	2	1
